# An uncommon cause of intermittent dysphagia 

**Published:** 2021

**Authors:** Saeed Abdi, Mehran Mahdavi Roshan, Naghmeh Salarieh

**Affiliations:** *Gastroenterology and Liver Diseases Research Center, Research Institute for Gastroenterology and Liver Diseases, Shahid Beheshti University of Medical Sciences, Tehran, Iran *

## Introduction

 A 42-year-old woman was admitted to the gastrointestinal department of Taleghani Hospital, a teaching referral hospital in Tehran, Iran, with epigastric pain, fever, and dysphagia. She complained of intermittent dysphagia, regurgitation, and epigastric pain (with no benefit from PPI treatment) one month in each year from five years before admission.

The patient was married and resided in Tehran, Iran. She had no history of smoking, opium addiction, alcohol consumption, or consumption of any medications. She had no family history of gastrointestinal cancers or problems similar to hers. From five years ago, she had experienced dysphagia and pain in the throat and epigastric area which had even been treated with the diagnosis of thyroiditis in the first year of her symptoms. Her BP = 110/70, HR = 98, and T = 37.7 at the time of admission. Hemoglobin was the only abnormal laboratory finding (Hb = 10) that could be explained by minor thalassemia.

**Figure 1:**
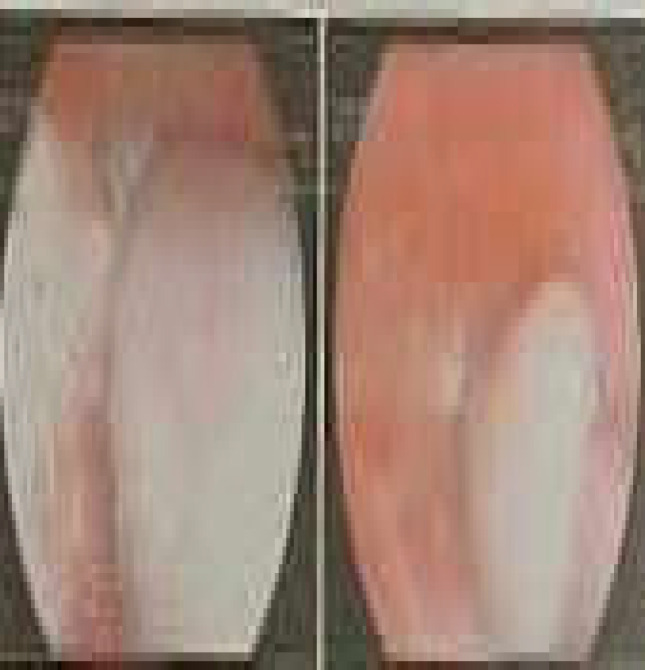
Upper GI endoscopy. The scope could not passed because of an esophageal lesion presence in 20-25 cm from incisura

An upper GI endoscopy was done for the patient, but the scope could not passed because of an esophageal lesion presence in 20-25 cm from incisura (Figure 1).

**Figure 2:**
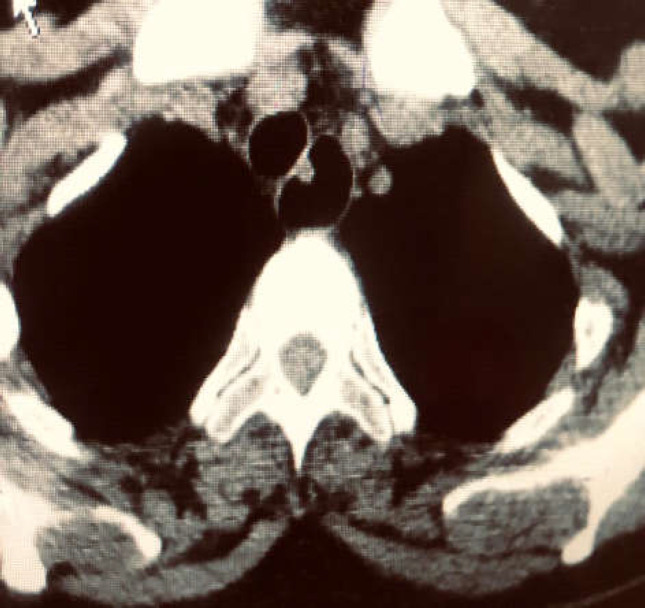
A mediastinal CT scan showed a non-enhancing lesion with well-defined margins in the middle to distal esophagus with no invasion or lymph node involvement

A mediastinal CT scan showed a non-enhancing lesion with well-defined margins in the middle to distal esophagus with no invasion or lymph node involvement (Figure 2).

**Figure 3:**
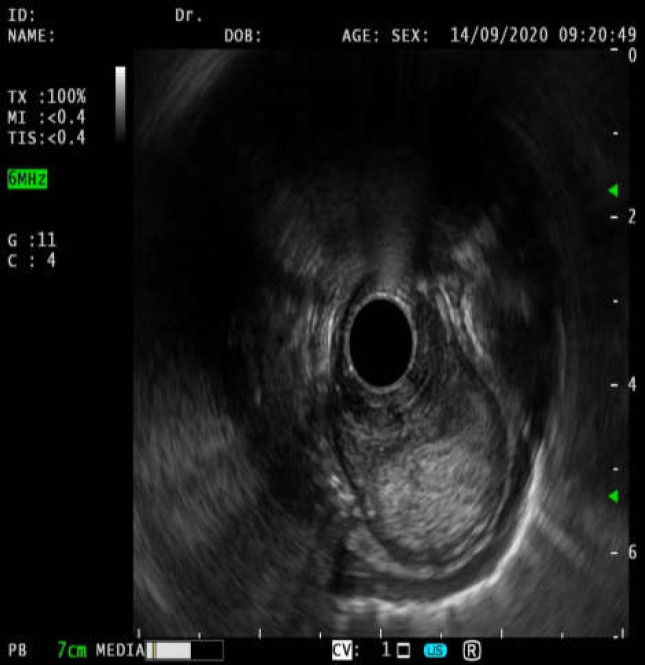
Endosonography report

Endosonography was another diagnostic procedure which showed one hypoechoic lesion with diameter of 46*25 mm and length of 13 mm (from 20 cm down to 33 cm of incisors) which contained lots of debris and was surrounded by a double layered wall (Figure 3). 

**What is your diagnosis?**

**What is the next step?**

## Discussion

Esophageal duplication cysts are usually asymptomatic gastrointestinal lesions that are infrequently seen and often diagnosed as a secondary finding (1).

Duplications can be found along the entire length of the esophagus with varying presentations depending on their location and compression effects on neighboring structures. The complicated duplications can present with life-threatening symptoms (2).

Esophageal duplication in adults is rare. Esophageal cyst is the second most common duplication of the GI tract (3).

Surgery has become the best treatment for this disease (1). On endoscopy, the cysts noticed as a protruding submucosal mass covered by normal mucosa. If duplication cysts are suspected based on endoscopy, EUS can confirm the diagnosis. A surgery consultation was requested for our patient, and she is going to have the operation this coming month.

## Conflict of interests

The authors declare that they have no conflict of interest.
